# The study of aqueous extract of *Pterocarpus marsupium* Roxb. on cytokine TNF-α in type 2 diabetic rats

**DOI:** 10.4103/0253-7613.71922

**Published:** 2010-12

**Authors:** Kirana Halagappa, H.N. Girish, B.P. Srinivasan

**Affiliations:** Delhi Institute of Pharmaceutical Sciences and Research (DIPSAR), M. B. Road, Sector-III, Pushp Vihar, New Delhi - 110 017, India; 1 T.V.M. College of Pharmacy, Gandhinagar, Bellary - 583 103, India; 2 Department of Pharmacology, Delhi Institute of Pharmaceutical Sciences and Research (DIPSAR), M.B. Road, Sector-III, Pushp Vihar, New Delhi - 110 017, India

**Keywords:** Cytokine, *Pterocarpus marsupium*, TNF-α, Type 2 diabetes

## Abstract

**Objective:**

This study was designed to investigate the effect of aqueous extract of *Pterocarpus marsupium* Roxb. on elevated inflammatory cytokine, tumor necrosis factor (TNF)-α in type 2 diabetic rats.

**Materials and Methods:**

Type 2 diabetes was induced by administering streptozotocin (90 mg/kg, i.p.) in a neonatal rat model. Aqueous extract of *P. marsupium* at a dose of 100 and 200 mg/kg was given orally to desired group of animals for a period of 4 weeks. After 4 weeks of drug treatment, parameters such as fasting blood glucose, postprandial blood glucose, and TNF-α in serum were analyzed.

**Results:**

Aqueous extract of *P. marsupium* at both doses, i.e., 100 and 200 mg/kg, decreased the fasting and postprandial blood glucose in type 2 diabetic rats. The 200 mg/kg had more pronounced effect on postprandial hyperglycemia. The drug also improved the body weight of diabetic animals. Cytokine TNF-α was found to be elevated in untreated diabetic rats due to chronic systemic inflammation. The aqueous extract at both doses significantly (*P* < 0.001) decreased the elevated TNF-α level in type 2 diabetic rats.

**Conclusion:**

Modulation of cytokine TNF-α by the rasayana drug *P. marsupium* is related with its potential anti-diabetic activity.

## Introduction

Elevated circulating inflammatory cytokines such as tumor necrosis factor (TNF)-α, interleukin (IL)-1β, and IL-6 are observed in patients with postprandial hyperglycemia.[1,2] Activated innate immune system and chronic systemic inflammation are an early process in the pathogenesis of type 2 diabetes.[3] A variety of stressors such as infection, tissue injury and food cause macrophages, adipocytes, endothelial cells, etc., to secrete inflammatory cytokines.[4] Cytokines are the small soluble peptides released by the cells of immune system to communicate and influence their function. Cytokine namely TNF-α has been implicated in insulin resistance.[5] TNF-α stimulates the endothelial production of adhesion molecules such as E-selectin and vascular cell adhesion molecule-1 (VCAM-1).[6] E-selectin and VCAM-1 accelerate the atherosclerosis and vascular complications in diabetes.[7] In this view, development of a drug which modulates the cytokine TNF-α in type 2 diabetes would be a novel approach in early intervention of the disease.

*Pterocarpus marsupium* Roxb. (Leguminosae) is a plant drug belonging to the group called rasayana in ayurvedic system of medicine.[8] Rasayana drugs are immunomodulators and relieve stress in the body.[9] In ayurveda, aqueous extract of heart-wood of *P. marsupium* is used in treatment of diabetes.[10] Although there are several reports on *P. marsupium* as an anti-diabetic drug,[11–13] there is no focus on the relevance of its rasayana property and anti-diabetic activity. Therefore, the present investigation was designed to study the action of aqueous extract of *P. marsupium* on TNF-α activity in type 2 diabetic rats.

## Materials and Methods

### Collection and authentication of plant material

Heart-wood of *P. marsupium* Roxb. was collected from local market of Udupi located in Karnataka state, India, during the month of November-December. It was dried under shade at temperature not exceeding 40 °C. Drug sample was authenticated and deposited (Voucher number: Wood/2006/745/62) at Department of Raw Materials Herbarium and Museum, National Institute of Science Communication and Information Resources (NISCAIR), New Delhi.

### Preparation of aqueous extract

Dried heart-wood was grounded into a moderately coarse powder (# 22) in domestic electric grinder. One part of the powdered drug was boiled with 16 parts of water for a period of 15 min and filtered hot through muslin cloth. Filtrate was then lyophilized by continuous freeze drying process for 36 h. The freeze drier (Allied Frost SZ 7510, New Delhi, India) was maintained at the temperature of −50 °C and pressure of 0.030–0.038 Torr during the entire operation. The dried aqueous extract (6.8%) was packed in air-tight container and stored in a desiccator at room temperature for further studies.[10,14] A preliminary phytochemical analysis of the aqueous extract showed the presence of carbohydrates, tannins, flavonoids, and polyphenolic compounds.

### Dose and drug solution

According to earlier reports, *P. marsupium* was found to be non-toxic up to 8 g/kg in albino mice.[15] The effective dose of the drug varied from 100 to 250 mg/kg in rats.[16] Hence, the present study was carried at two dose levels, i.e., at 100 and 200 mg/kg body weight. To prepare the test drug, required quantity of the aqueous extract of *P. marsupium* was dissolved in distilled water to have a desired dose in 1 mL solution.

### Animals

Wistar albino rats (140–160 g) of either sex were housed under standard laboratory conditions at temperature 25 ± 2 °C and 55 ± 5% relative humidity with a regular 12 h light:12 h dark cycle. Animals were given standard rat pellet diet and tap water ad libitum. The study protocol (Protocol number: 06/DIPSAR/IAEC/2004) was approved by Institutional Animal Ethical Committee (IAEC), Delhi Institute of Pharmaceutical Sciences and Research (DIPSAR), New Delhi.

### Streptozotocin-induced neonatal rat model for type 2 diabetes

Type 2 diabetes was induced by administering streptozotocin (90 mg/kg i.p.) in 2-day-old neonatal rats. After 6 weeks of streptozotocin injection, rats showing the fasting blood glucose more than 160 mg/dL were considered as type 2 diabetes positive.[17]

### Experimental groups

Wistar albino rats of either sex were randomly allotted into five groups of six animals each. Equal number of males and females were maintained in each group and caged separately. Group I served as normal and received distilled water. Group II served as type 2 diabetic control and received distilled water. Group III was type 2 diabetic treated with 100 mg/kg of aqueous extract of *P. marsupium*. Group IV was type 2 diabetic treated with 200 mg/kg of aqueous extract of *P. marsupium*.[18] Group V was type 2 diabetic treated with 10 mg/kg of gliclazide. Drug treatment was given each morning with the help of oral catheter for a period of 4 weeks. Body weight was determined at the end of every week. After 4 weeks of drug treatment, parameters such as fasting blood glucose, postprandial blood glucose, and TNF-α in serum were analyzed.

### Estimation of fasting blood glucose

Blood samples were withdrawn from overnight fasted animals by retro-orbital puncture under mild ether anesthesia and centrifuged at 3000 rpm for 15 min, at 4 °C in cooling centrifuge (Remi, C-24 BL, Mumbai, India). Glucose in serum was estimated by glucose oxidase and peroxidase (GOD-POD kit) method. Intensity of the red quinoneimine was measured at 540 nm in Autoanalyzer (Logotech, Tecno 168, Italy).[19]

### Estimation of postprandial blood glucose

Blood samples were withdrawn from overnight fasted animals for basal reading (0 min). Then, the animals were treated with respective drug solutions. Thirty minutes after the drug treatment, glucose solution at a dose of 2.5 g/kg body weight was administered orally with the help of oral catheter.[20] Blood samples were withdrawn after 120 min of oral glucose load (postprandial). Glucose in serum was estimated by glucose oxidase and peroxidase (GOD-POD kit) method.

### Estimation of TNF-α

TNF-α in serum was estimated by ELISA (Rat TNF-α ELISA KIT, DIACLONE, France). Sufficient microwell strips were taken out of the pouch. Standard diluents, 100 μL, and serum, 100 μL, were added into the blank and sample well, respectively. Then, 50 μl of diluted biotinylated anti-rat TNF-α was added to all the wells. Wells were incubated for 3 h at 37 °C. Plate was removed and liquid from the wells were aspirated and 0.3 mL of washing solution was added into each well and aspirated. Washing was repeated two more times. Then, 100 μL of streptavidin-HRP solution was added to all the wells including blank. The wells were incubated at 37 °C for 30 min and 0.3 mL of washing solution was added into each well and aspirated. Washing was repeated two more times. A total of 100 μL of chromogen-TMB (substrate) solution was added to all the wells including blank. The wells were again incubated at 37 °C for 15 min. The enzyme substrate reaction was stopped by quickly adding 100 μL of sulfuric acid. Absorbance of the color developed in the wells was measured at 420 nm in ELISA reader (Awareness Technology, Mumbai, India).[21] TNF-α in the sample was analyzed from the standard curve plotted with a limit of detection, 20 pg/mL.

### Statistical analysis

Data are expressed as mean ± SEM. Statistical comparison between different groups was done using one-way analysis of variance (ANOVA) followed by Tukey-Kramer multiple comparison test. *P* < 0.05 was considered as statistically significant.

## Results

### Effect on fasting blood glucose

**Figure 1:**
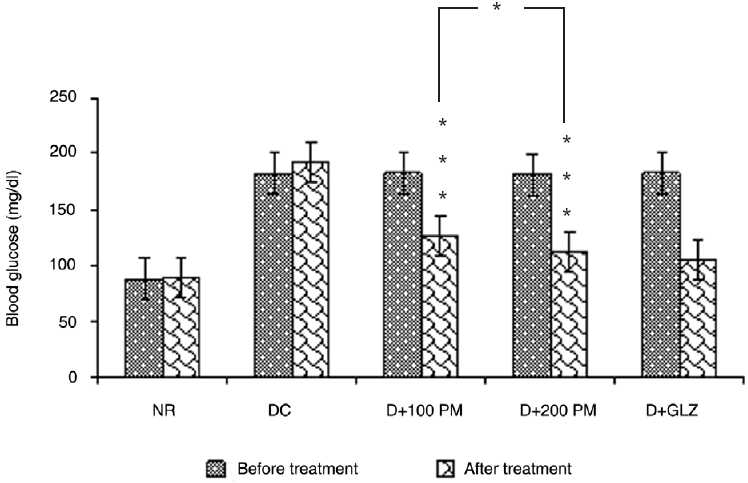
Effect of aqueous extract of *Pterocarpus marsupium* (PM) on fasting blood glucose of type 2 diabetic rats. Values are mean ± SEM; n=6; *P < 0.05; ***P < 0.001 as compared to type 2 diabetic control; F = 165.5; df = 4, 25 (one-way ANOVA followed by Tukey-Kramer multiple comparison test); NR: Normal, DC: type 2 diabetic control, D + 100 PM: type 2 diabetic treated with 100 mg/kg of PM, D + 200 PM: type 2 diabetic treated with 200 mg/kg of PM, D + GLZ: type 2 diabetic treated with gliclazide.

Fasting blood glucose of type 2 diabetic rats was found to be 182.5 ± 4.1 mg/dL. Aqueous extract of *P. marsupium* at both doses, i.e. 100 mg/kg and 200 mg/kg significantly (*P* < 0.001) decreased the fasting blood glucose in type 2 diabetic rats. However, the difference between the effect produced by the two doses, i.e. 100 mg/kg and 200 mg/kg was significant (*P* < 0.05) when analyzed for inter-group comparison. Gliclazide used as the standard drug of comparison significantly (*P* < 0.001) decreased the fasting blood glucose as compared to type 2 diabetic control group [Figure 1].

### Effect on postprandial blood glucose

**Figure 2:**
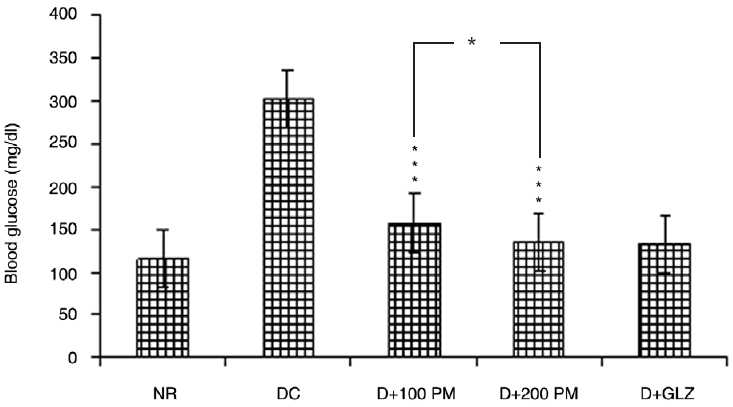
Effect of aqueous extract of *Pterocarpus marsupium* (PM) on postprandial blood glucose of type 2 diabetic rats. Values are mean ± SEM; n = 6; *P < 0.05; ***P < 0.001 as compared to type 2 diabetic control; F = 237.3; df = 4, 25 (one-way ANOVA followed by Tukey- Kramer multiple comparison test). NR: Normal, DC: type 2 diabetic control, D + 100 PM: type 2 diabetic treated with 100 mg/kg of PM, D + 200 PM: type 2 diabetic treated with 200 mg/kg of PM, D + GLZ: type 2 diabetic treated with gliclazide.

At 120 min after the oral glucose load (postprandial), blood glucose of normal rats was found to be 112.3 ± 2.8 mg/dL. In the case of type 2 diabetic control group, the postprandial blood glucose was 301.4 ± 5 mg/dL. Aqueous extract of *P. marsupium* at 100 mg/kg and 200 mg/kg dose decreased the postprandial hyperglycemia significantly (*P* < 0.001) as compared to diabetic control group. Difference between 100 mg/kg and 200 mg/kg dose was significant (*P* < 0.05) when analyzed for inter-group comparison. Gliclazide decreased the postprandial hyperglycemia significantly (*P* < 0.001) [Figure 2].

### Effect on body weight

**Figure 3:**
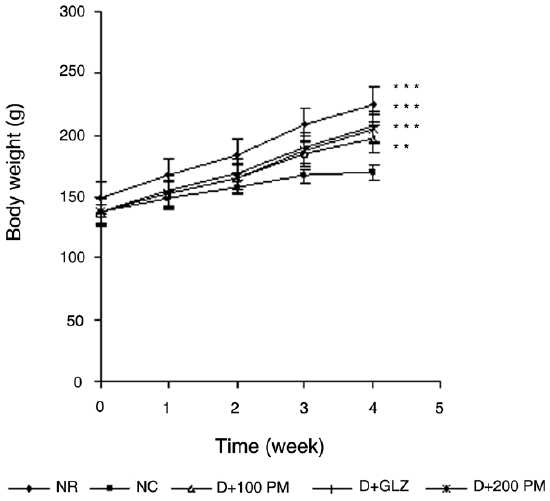
Effect of aqueous extract of *Pterocarpus marsupium* (PM) on body weight of type 2 diabetic rats. Values are mean ± SEM; n=6; **P < 0.01, ***P < 0.001 as compared to type 2 diabetic control; F = 17.8; df = 4, 25 (one-way ANOVA followed by Tukey-Kramer multiple comparison test). NR: Normal, DC: type 2 diabetic control, D + 100 PM: type 2 diabetic treated with 100 mg/kg of PM, D + 200 PM: type 2 diabetic treated with 200 mg/kg of PM, D + GLZ: type 2 diabetic treated with gliclazide.

Two weeks of drug treatment did not improve the body weight of diabetic rats. By the end of third week, 100 mg/kg and 200 mg/kg of aqueous extract of *P. marsupium* increased the body weight significantly (*P* < 0.05 and *P* < 0.01, respectively), as compared to diabetic control group. By the end of fourth week, 100 mg/kg and 200 mg/kg showed significant (*P* < 0.01 and *P* < 0.001, respectively) increase in body weight. Gliclazide significantly (*P* < 0.001) improved the body weight of diabetic rats. Body weight of various experimental groups at basal level, i.e. before drug treatment and at the end of 1, 2, 3, and 4 weeks of drug treatment is shown in Figure 3.

### Effect on TNF-α

**Figure 4:**
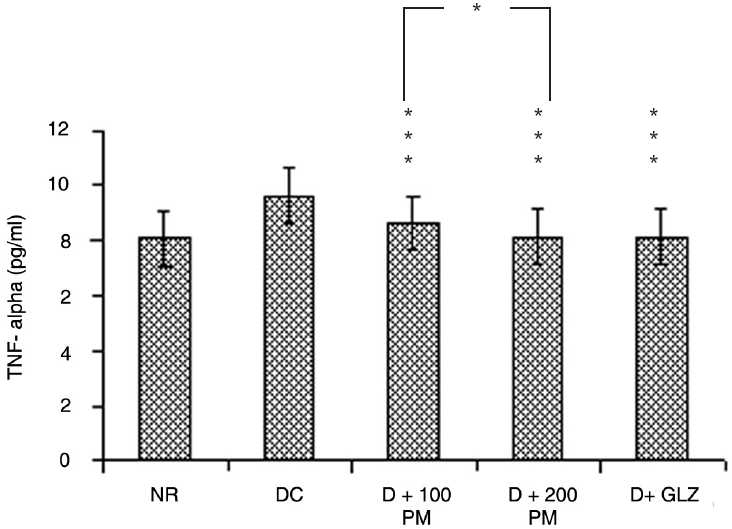
Effect of aqueous extract of *Pterocarpus marsupium* (PM) on TNF-α level in type 2 diabetic rats. Values are mean ± SEM; n = 6; *P < 0.05; ***P < 0.001 as compared to type 2 diabetic control; F = 32.8; df = 4, 25 (one-way ANOVA followed by Tukey-Kramer multiple comparison test). NR: Normal, DC: type 2 diabetic control, D + 100 PM: type 2 diabetic treated with 100 mg/kg of PM, D + 200 PM: type 2 diabetic treated with 200 mg/kg of PM, D + GLZ: type 2 diabetic treated with gliclazide.

TNF-α was found be elevated in type 2 diabetic rats as compared to normal group. Aqueous extract of *P. marsupium* at 100 mg/kg and 200 mg/kg dose significantly (*P* < 0.001) decreased the elevated TNF-α in type 2 diabetic rats. Difference between 100 and 200 mg/kg dose is statistically significant (*P* < 0.05) when analyzed for inter-group comparison. Drug at higher dose, i.e. 200 mg/kg, had more pronounced effect on elevated TNF-α. Gliclazide also significantly (*P* < 0.001) decreased the elevated TNF-α in type 2 diabetic rats [Figure 4].

## Discussion

Aqueous extract of *P. marsupium* at 100 mg/kg and 200 mg/kg dose had significant effect on both fasting and postprandial blood glucose in type 2 diabetic rats. Postprandial hyperglycemia is an earliest metabolic abnormality to occur in type 2 diabetes. This state initiates the development of microvascular and macrovascular complications.[22] Most of the currently available anti-diabetic therapies reduce the fasting blood glucose but have a little impact on postprandial hyperglycemia.[23] In this view, *P. marsupium* at a dose of 200 mg/kg could be a better drug in treatment of type 2 diabetes. Reduction in blood glucose may be mediated through enhanced insulin secretion by regeneration of β-cells of islets of Langerhans.[24,25] Since rasayana drugs are rejuvenators,[9] there may be regeneration of pancreas. Aqueous extract of heart-wood of *P. marsupium* is rich in flavonoids and polyphenols.[14] Antioxidant potential of flavonoids coupled with their nutritional value may be responsible for rejuvenation.

Cytokine TNF-α has been implicated in insulin resistance.[5,6] Insulin resistance is a primary metabolic defect in type 2 diabetes. Binding of insulin to its receptor inducing autophosphorylation at multiple tyrosine sites is a key element in insulin signaling pathway. Activated receptor further phosphorylates insulin receptor substrate (IRS). Finally insulin signals are transduced from IRS to major pathway of intracellular serine-threonine kinase namely phosphatidyl inositol (PI) 3-kinase. PI 3-kinase stimulates the translocation of glucose transporters GLUT-4 from intracellular pool to cell membrane for uptake of glucose by the cell.[26] Cytokine TNF-α has direct inhibitory effect on tyrosine kinase and phosphorylation cascade of insulin signaling pathway.[3] TNF-α mediates insulin resistance also through indirect effects including increasing free fatty acids in circulation, stimulation of insulin counter-regulatory hormones, impairment of endothelial function, or inhibiting the glucose-stimulated insulin release by pancreatic β-cells.[27] The above interference of TNF-α in various pathways is justified by its elevated levels in type 2 diabetic rats. Elevated TNF-α indicates the activated innate immune system followed by chronic systemic inflammation associated with type 2 diabetes. Decrease in elevated TNF-α by the aqueous extract of *P. marsupium* along with its blood glucose lowering effect suggests that the immunomodulatory property of this rasayana drug could be related with its potential anti-diabetic activity.

Body weight of type 2 diabetic rats was found to be less during the course of development as compared to normal animals. Elevated TNF-α inhibits the uptake of free fatty acids from circulation and accelerates the lipolysis in adipose tissue, leading to weight loss in type 2 diabetes. Paracrine effect of TNF-α is high in obesity and type 2 diabetes.[27] Weight loss in diabetes is also generally due to continuous excretion of glucose from the body. Long-term presence of TNF-α has an appetite suppressing effect. Improved body weight of the drug-treated animals seems to be due to TNF-α modulation.

Rasayana drugs are effective by modulating the release of cytokines.[28] Rasayana drugs namely Boerhaavia diffusa, Picrorhiza kurroa, Tinospora cordifolia, etc. are reported to suppress the release of TNF-α from macrophages.[9,29] Modulation of cytokine TNF-α by the aqueous extract of *P. marsupium* is possibly due to flavonoids present in the drug. Many flavonoids have inhibitory effect on TNF-α.[30] As natural modulators of pro-inflammatory gene expression, flavonoids are considered as potential candidates for new immunomodulatory agents.

Cytokine TNF-α has been reported to down regulate the peroxisome proliferators activator receptor (PPAR)-γ expression.[31] PPARs are the class of nuclear receptors that co-ordinately regulates the expression of large gene array and modulates the important metabolic events of cell. Isoflavone from *P. marsupium* has upregulated the PPAR-γ; gene expression *in-vitro* cultured L6 myotubes.[32] In this study, modulation of cytokine TNF-α by the aqueous extract of *P. marsupium* has indirect effect on PPAR-γ; expression. By decreasing TNF-α, drug can up-regulate the PPAR-γ; and in turn the glucose metabolism.

## Conclusion

*P. marsupium* modulates the inflammatory cytokine TNF-α in type 2 diabetic rats. Drug at 200 mg/kg dose has more pronounced effect. Rasayana property of *P. marsupium* is related with its potential anti-diabetic activity.
